# Lobar pneumonia caused by Ralstonia pickettii in a sixty-five-year-old Han Chinese man: a case report

**DOI:** 10.1186/1752-1947-5-377

**Published:** 2011-08-15

**Authors:** Wensen Pan, Zhiming Zhao, Mei Dong

**Affiliations:** 1 Department of Respiratory Medicine, the Second Hospital of Hebei Medical University, Shijiazhuang, China; 2 Department of Reproductive Medicine, the Second Hospital of Hebei Medical University, Shijiazhuang, China; 3 Department of Internal Medicine, the Affiliated Hospital of Hebei University of Science and Technology, Shijiazhuang, China

## Abstract

**Introduction:**

*Ralstonia pickettii *is a gram-negative, oxidase-positive bacillus and is an emerging pathogen found in infections described in hospital settings. The cases reported in the literature mostly are nosocomial infections due to contaminated blood products, sterile water, saline, treatment fluids and venous catheters. Human infection unrelated to contaminated solutions is rare. We report a case of lobar pneumonia and pulmonary abscess caused by *Ralstonia pickettii *in an older patient.

**Case presentation:**

A sixty-five-year old Han Chinese man presented having had cough, expectoration, chest pain and fever lasting for twenty days. His medical history was notable for hypertension over the previous ten years, and the habit of smoking for forty years. A thoracic computed tomography scan supported the diagnosis of right-sided lobar pneumonia. A lung biopsy was done and pathological analysis confirmed lobar pneumonia. Two lung biopsy specimens from separate sites grew *Ralstonia pickettii*. After six days, a repeat thoracic scan revealed a right-sided abscess. A thoracentesis was performed and the purulent fluid grew *Ralstonia pickettii*. The chest tube remained inserted to rinse the cavity with sterile sodium chloride. He received an antibiotic course of intravenous cefoperazone sodium-sulbactam sodium for eighteen days and imipenem-cilastatin for twelve days. A repeat chest X-ray revealed resolution of the pulmonary abscess and improvement of pneumonia. He remained afebrile and free of respiratory symptoms after treatments.

**Conclusion:**

This case demonstrates a *Ralstonia pickettii *infection in the absence of an obvious nosocomial source. It is possible that such cases will become common in the future. Therefore, further studies are needed to evaluate its sensitivity to common antibiotics.

## Introduction

*Ralstonia pickettii *(*R. pickettii*) is an emerging pathogen. It is ubiquitous in nature and is found naturally in soil and groundwater. *R. pickettii *was first isolated in 1973 and included in the genus *Pseudomonas *[1]. The rod-shaped organism was reclassified in the *Burkholderia *and then the *Ralstonia *genera, receiving its current name in 1995 [2]. *R. pickettii *is often confused with other similar bacteria, increasing the difficulty of classifying and treating this pathogen.

*R. pickettii *can be isolated from various clinical specimens, such as sputum, blood, infected wounds, urine, ear, nose swabs, and cerebrospinal fluid. It is also commonly found in the respiratory tract secretions of cystic fibrosis patients. Most infections with *R. pickettii *are seen in the hospital setting resulting in bacteremia and/or septicemia and respiratory infections and/or pneumonia [3]. The cases reported in the literature are mostly nosocomial infections due to contaminated solutions including blood products, sterile water, saline, chlorhexidine solution, treatment fluids for the respiratory tract, and contaminated venous catheters [4-7]. Human infection unrelated to contaminated solutions is rare. There is only one documented case of an empyema caused by *R. pickettii*, and our case is similar in some respects [8]. Our case is perhaps the first one reported of a lobar pneumonia and pulmonary abscess caused by *R. pickettii*.

## Case presentation

A sixty-five-year old Han Chinese man presented with cough, expectoration, chest pain and fever lasting twenty days to the emergency room. His medical history was notable for hypertension over the previous ten years, and a forty-year smoking history (20 cigarettes per day). He did not have previous exposure to respiratory therapy solutions and had not taken any antimicrobial agents in the past five years. Pleuritic chest pain was the most prominent symptom. The cough was dry initially, but about five days later became productive. He had a sudden onset of a high fever to 39°C, which resulted in rigors. Upon initial presentation, he received antibiotic therapy of intravenous penicillin sodium for seven days, lavo-ofloxacin for five days and cefotaxime sodium for seven days in turn, but his symptoms did not improve, resulting in admission to our hospital.

**Figure 1:**
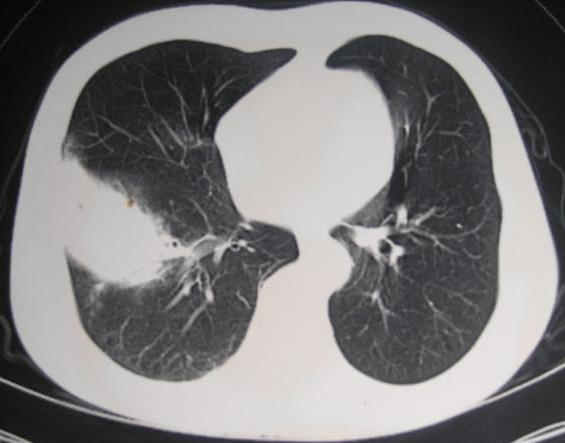
**Computed tomography scan (GE Medical System. lightspeed 16) of the thorax showing features of lobar pneumonia**. In this image right lung lower lobe soft tissue density shadow can be seen.

**Figure 2:**
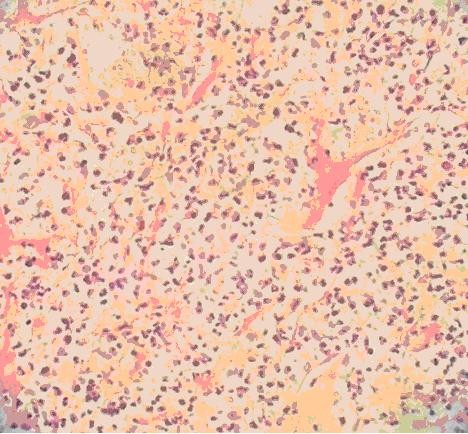
**Lung biopsy appearance of lobar pneumonia-like changes of the gray phase of liver**. It is showing that alveolar space is clearly visible, a large number of cellulose can be seen seeping into cavity to form a network and through Trichoderma Kong mutual links with the neighboring alveolar space. (hematoxylin and eosin, magnification ×40).

**Figure 3:**
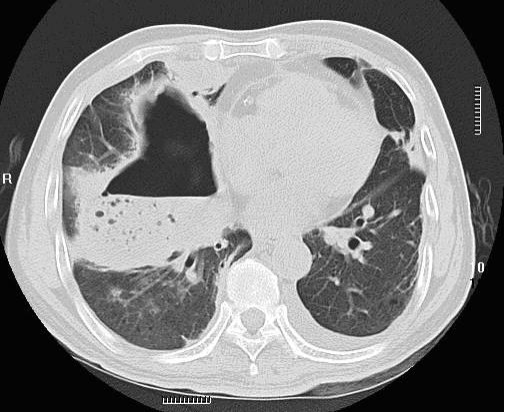
**Computed tomography scan of the thorax**. The scan shows features typical of pulmonary abscesses, consolidation with a single cavity containing an air-fluid level in the right lung after six days of intravenous cefepime treatment.

His initial physical examination upon admission revealed a temperature of 38.8°C; blood pressure, 120/85 mmHg; respiratory rate, 23 breaths per minute; and pulse, 90 beats per minute. Evaluation for a source of the fever demonstrated a right-sided lobar pneumonia on chest X-ray. A thoracic computed tomography (CT) scan supported the diagnosis of right-sided lobar pneumonia (Figure 1). Subsequently, a CT-guided lung biopsy was done, which confirmed it to be lobar pneumonia in the period of gray hepatization (Figure 2). Two biopsy specimens from separate sites grew *R. pickettii *in pure cultures, which was identified by the API 20NE system (bioMérieux France, No.0041445). He received an antibiotic course of intravenous cefepime for six days. A repeat thoracic CT scan revealed the presence of a right-sided abscess (Figure 3). A centesis was performed and the purulent fluid grew *R. pickettii*. The chest tube placed for drainage remained in the cavity of the abscess for rinsing with sterile sodium chloride solution. Because the pathogen had not responded to two antibiotic treatments, its antimicrobial susceptibilities were studied by the disk diffusion method of the Clinical and Laboratory Standards Institute (CLSI) (9). The breakpoints used to determine resistance and susceptibility to the antibiotics were provided simultaneously (Table 1). In this case, the *R. pickettii *isolate was susceptible to cefoperazone sodium-sulbactam sodium, ceftazidime, and imipenem according to the disk diffusion method. The pathogen was resistant to amikacin, ceftriaxone, ciprofloxacin, mezlocillin, aztreonam, and gentamicin. Our patient received an antibiotic course of intravenous cefoperazone sodium-sulbactam sodium for eighteen days and imipenem-cilastatin for twelve days. A repeat chest X-ray performed forty-eight days later revealed resolution of the pulmonary abscess and improvement of pneumonia. The patient remained afebrile and free of respiratory symptoms at follow-up two months later.

**Table 1:** Breakpoints used to determine resistance and susceptibility to antimicrobial therapy

Antimicrobial Agent	Disk Content	Zone Diameter Breakpoints, nearest whole mm		
		**S**	**I**	**R**

mezlocillin	75 μg	≧ 16	-	≦ 15

ceftazidime	30 μg	≧ 18	15-17	≦ 14

cefoperazone	75 μg	≧ 21	16-20	≦ 15

ceftriaxone	30 μg	≧ 21	14-20	≦ 13

imipenem	10 μg	≧ 16	14-15	≦ 13

aztreonam	30 μg	≧ 22	16-21	≦ 15

gentamicin	10 μg	≧ 15	13-14	≦ 12

ciprofloxacin	5 μg	≧ 21	16-20	≦ 15

## Discussion

Human infection with *R. pickettii *without exposure to contaminated solutions is rare and isolation of the organism in culture alone is often attributed to laboratory contamination rather than to infection. Therefore, infection with *R. pickettii *is typically diagnosed when treatment targeting the organism and/or removal of an infected source is associated with clinical improvement. For example, a recently reported case of *R. pickettii *infection in a pediatric oncology unit described clinical improvement only with catheter removal and appropriate antimicrobial therapy [10]. In this case, isolation of *R. pickettii *in culture from a sterile site coupled with clinical improvement following thoracentesis and targeted antimicrobial therapy increases the likelihood that the organism was the pathogenic source. In our case, there had been no use of respiratory therapy solutions excluding the possibility of exposure to fluids contaminated with *R. pickettii*.

*R. pickettii *is generally believed not to be the primary pathogen and, alone, its infectivity is very low. Recent reports show that it can lead to a number of potentially serious infections, nosocomial outbreaks [4,11] and even death [3]. Antimicrobial susceptibility patterns reported for *R. pickettii *vary widely. *R. pickettii *can produce extended-spectrum β-lactamases, which are not commonly sensitive to inhibitors of β-lactamase [12-15]. They show that the organism is resistant in different degrees to ciprofloxacin, trimethoprim-pyrimidine, sulfamethoxazole, piperacillin-tazobactam, imipenem and cilastatin, ceftazidime. Following susceptibility studies, our patient was successfully treated with intravenous cefoperazone sodium-sulbactam sodium for eighteen days and imipenem-cilastatin for twelve days.

## Conclusion

We describe the case of an older man who developed *R. pickettii *infection in the absence of an obvious nosocomial source demonstrating the possibility that such *de novo *cases will become more common in the future. Although it is of low virulence, it has been identified as causing many potentially harmful infections, and even death. The pathogen was resistant to many antibiotics, so its sensitivity to the common antibiotics should be monitored regularly.

## Consent

Written informed consent was obtained from the patient for publication of this case report and accompanying images. A copy of the written consent is available for review by the Editor-in-Chief of this journal.

## Abbreviations

CLSI: Clinical and Laboratory Standards Institute; CT: computed tomography; *R. Pickettii: Ralstonia pickettii*.

## Competing interests

The authors declare that they have no competing interests.

## Authors' contributions

WP collected the patient data and was a major contributor in writing the manuscript. ZZ and MD performed CT-guided lung biopsy and the histological examination of the lung. All authors read and approved the final manuscript.
